# A Narrative Review on the Monkeypox Virus: An Ongoing Global Outbreak Hitting the Non-Endemic Countries

**DOI:** 10.7759/cureus.43322

**Published:** 2023-08-11

**Authors:** Hira Nisar, Omer Saleem, FNU Sapna, Sunder Sham, Raja Sandeep Perkash, Nfn Kiran, FNU Anjali, Ansa Mehreen, Bebu Ram

**Affiliations:** 1 Nephrology, Sindh Institute of Urology and Transplantation, Karachi, PAK; 2 Medicine, Jinnah Postgraduate Medical Centre, Karachi, PAK; 3 Otolaryngology, Jinnah Postgraduate Medical Centre, Karachi, PAK; 4 Pathology, Montefiore Medical Center, Wakefield Campus, New York, USA; 5 Pathology and Laboratory Medicine, Lenox Hill Hospital, New York City, USA; 6 Research, Montefiore Medical Center, Wakefield Campus, New York, USA; 7 Pathology, Staten Island University Hospital, New York, USA; 8 Internal Medicine, Sakhi Baba General Hospital, Pano Akil, PAK; 9 Pathology and Laboratory Medicine, University of Chicago Pritzker School of Medicine, Evanston, USA; 10 Pathology, University at Buffalo, Buffalo, USA

**Keywords:** orthopox virus, dna virus, zoonosis, review, outbreak, infection, monkeypox

## Abstract

Monkeypox is a rare zoonotic DNA with lineage from the Poxviridae family, Chordopoxvirinae subfamily, and Orthopoxvirus genus. With a previous history of controlled and contained occasional outbreaks of the virus, currently, a widely erupted outbreak of monkeypox with progressively rising numbers has been reported since May 2022 in multiple countries of the western hemisphere that are not historically endemic for this infection, particularly the United Kingdom and European Union countries. We have written a comprehensive review article to help clinicians better understand the disease. The global cessation of smallpox vaccination has been hypothesized to cause the rise in monkeypox infections in recent years. Monkeypox, like any other viral infection, commences with prodromal symptoms; a maculopapular rash with centrifugal distribution usually follows. Polymerase chain reaction (PCR) confirms the diagnosis. Transmission in humans is possible through infected animals or humans. In the ongoing 2022 outbreak, the monkeypox virus has been undergoing novel mutations at an alarming rate. Treatment options for monkeypox are an area that still requires extensive research, and the utility of certain antiviral medications in treating monkeypox infection is currently being explored but is still controversial and debatable.

## Introduction and background

Monkeypox is a rare zoonotic deoxyribonucleic acid (DNA) virus belonging to the same family as smallpox, the Poxviridae family, Chordopoxvirinae subfamily, and Orthopoxvirus genus [1]. The virus was first isolated from monkeys at a laboratory in Copenhagen, Denmark, in 1958, hence the name, but some rodents and non-human primates can also act as natural reservoirs of this virus [2,3]. The first ever case of monkeypox in humans was reported in an infant in the Democratic Republic of Congo in 1970 [4]. Since then, small outbreaks have frequently occurred in Central and West Africa, which are historically endemic areas, while regions outside Africa are categorized as non-endemic areas for monkeypox infection. Two main genetic clades of monkeypox have been distinguished to date, namely, the Central African clade and the West African clade, the former manifesting more severe disease progression and a relatively higher mortality rate (10.6% and 3.6%, respectively). Monkeypox infections have been on the rise in the last few years. Only about 10 cases of monkeypox had been reported from 1978 to 2017 in West Africa, with no case being reported from Nigeria during this time until the reemergence of the West African clade was observed as a major outbreak in 2017 in Nigeria, with a total of 188 cases (146 suspected, 42 confirmed) [5-7].

A massive shift in epidemiological trends has been seen after the year 2000; between the years 2010 and 2019, the average age of presentation increased from four years to 21 years. The first monkeypox outbreak outside the historically endemic areas of Africa occurred in 2003 in the United States of America (USA) by the West African clade, with around 71 confirmed and suspected cases. The outbreak was caused by some infected African rodents that were imported from Ghana to Texas and got domesticated with prairie dogs after arrival in the USA. This contact chain erupted a monkeypox outbreak in the USA, infecting a total of 81 humans, according to the Centers for Disease Control and Prevention (CDC) [8-11]. The second incidence of monkeypox cases outside Africa occurred when a person, with a travel history, tested positive for monkeypox in Israel in September 2018. This was the first ever case taken outside Africa by a human reservoir, followed by cases reported in the United Kingdom (UK) in September 2018, December 2019, May 2021, and May 2022; Singapore in May 2019; and the USA in July and November 2021. Since May 2022, monkeypox cases continue to be on the rise in historically non-endemic countries, particularly in Europe and the UK. The viral strains isolated during the ongoing outbreak show the West African clade, with most cases having a recent travel history to non-endemic areas. None of the cases had traveling history to the historically endemic areas, while two cases from the UK surprisingly had no travel history or outside contact. The infected individuals from this 2022 outbreak were mostly men, particularly men who had sex with men (MSM). Statistics from Madrid, Spain, between April 2022 and June 16, 2022, showed that 99% of confirmed monkeypox cases were men and 93% were MSM. In May 2022, the UK reported six cases, while Canada 22 cases in MSM [11-13]. 

We performed a narrative review of the available literature on the monkeypox virus. During the literature search, we found that the majority of articles highlighted only the recent epidemiological trends, and very few articles discussed all aspects of this infection. Therefore, we decided to write a comprehensive narrative review inclusive of other aspects as well, such as clinical manifestations, mode of transmission, prevention, and potential treatment options.

## Review

Epidemiology

According to the CDC website, a total of 88,549 confirmed cases of monkeypox, with 152 deaths, have been reported around the globe till July 19, 2023, 5:00 post meridiem Eastern daylight time (PM EDT), among which the United States is currently leading with 30,611 cases. A death toll of 152 out of 88,549 cases makes the global case fatality ratio (CFR) for monkeypox about 0.172% (rounded up to three decimal places). For a more detailed breakdown of locations, please see Table 1 [14].

**Table 1:** Global statistics of the current monkeypox outbreak, May 2022 onwards. The locations with asterisk sign * are historically endemic for monkeypox outbreaks (data as of July 19, 2023, 5:00 PM EDT) This table has been directly reproduced from the website of the Centers for Disease Control and Prevention (CDC) and does not require copyright permission as per the website [14].

Location	Cases	Deaths
Andorra	4	0
Argentina	1129	2
Aruba	3	0
Australia	145	0
Austria	328	0
Bahamas	3	0
Bahrain	2	0
Barbados	1	0
Belgium	795	2
Benin	3	0
Bermuda	1	0
Bolivia	265	0
Bosnia and Herzegovina	9	0
Brazil	10,967	16
Bulgaria	6	0
Cameroon*	41	3
Canada	1,496	0
Central African Republic*	30	1
Chile	1,442	3
China	114	0
Colombia	4,090	0
Costa Rica	225	0
Croatia	33	0
Cuba	8	1
Curaçao	3	0
Cyprus	5	0
Czechia	71	1
Democratic Republic of the Congo*	734	3
Denmark	196	0
Dominican Republic	52	0
Ecuador	557	3
Egypt	3	0
El Salvador	104	0
Estonia	11	0
Finland	42	0
France	4,147	0
Georgia	2	0
Germany	3,691	0
Ghana*	127	4
Gibraltar	6	0
Greece	88	0
Greenland	2	0
Guadeloupe	1	0
Guatemala	405	1
Guyana	2	0
Honduras	44	0
Hong Kong	10	0
Hungary	80	0
Iceland	16	0
India	22	1
Indonesia	1	0
Iran	1	0
Ireland	229	0
Israel	263	0
Italy	957	0
Jamaica	21	0
Japan	191	0
Jordan	1	0
Latvia	6	0
Lebanon	27	0
Liberia*	13	0
Lithuania	5	0
Luxembourg	57	0
Malta	34	0
Martinique	7	0
Mexico	4,039	30
Moldova	2	0
Monaco	3	0
Montenegro	2	0
Morocco	3	0
Mozambique	1	1
Nepal	1	0
Netherlands	1,265	0
New Caledonia	1	0
New Zealand	41	0
Nigeria*	843	9
Norway	96	0
Pakistan	5	0
Panama	237	1
Paraguay	126	0
Peru	3,812	20
Philippines	5	0
Poland	217	0
Portugal	965	1
Qatar	5	0
Republic of the Congo*	5	0
Romania	47	0
Russia	2	0
San Marino	1	0
Saudi Arabia	8	0
Serbia	40	0
Singapore	25	0
Slovakia	14	0
Slovenia	47	0
South Africa*	5	0
South Korea	124	0
Spain	7,559	3
Sri Lanka	4	0
Sudan	19	1
Sweden	260	0
Switzerland	554	0
Taiwan	231	0
Thailand	119	0
Turkey	12	0
Ukraine	5	0
United Arab Emirates	16	0
United Kingdom	3,761	0
United States of America	30,611	45
Uruguay	19	0
Venezuela	12	0
Vietnam	3	0

**Figure 1:**
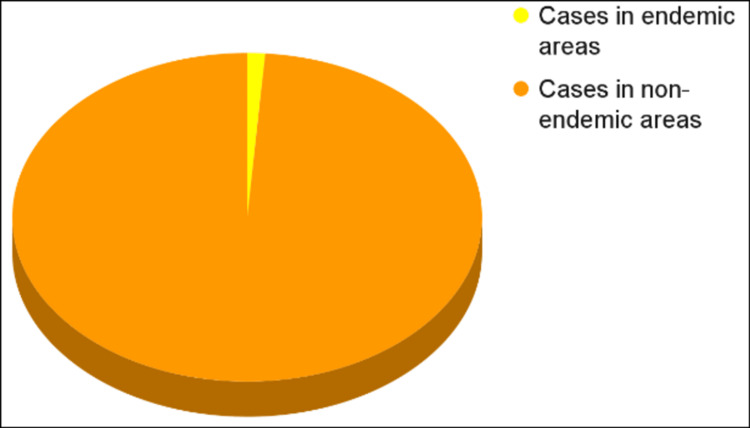
Global distribution of monkeypox cases in endemic and non-endemic areas: 97.975% of cases (86,756 out of 88,549) in non-endemic areas and 2.025% cases (1,793) in historically endemic areas This graphical representation is the authors' own creation, based on the statistics in Table 1 [14].

The proportion of monkeypox cases in endemic versus non-endemic areas of the globe is shown in Figure 1. In the current outbreak, from a total of 88,549 cases, exactly 86,756 cases (97.975%) have been reported in non-endemic locations for monkeypox. Meanwhile, only 1,793 (2.025%) have been reported in historically endemic areas [14].

Transmission

In the endemic parts of Africa, the monkeypox virus has survived through some rodents and mammals acting as natural reservoirs, such as squirrels, African giant pouched rats, striped mice, non-human primates (NHPs), and dormice with animal to human transmission, possibly consequential to mutations in viral strains [3,5,14,15-17]. Reservoir animals can transmit the virus to humans through direct or indirect exposure of humans with their saliva, blood, or other body secretions, inadequately cooked meat, or mucocutaneous lesions [5]. Transmission among humans is also possible by inhalation of infected respiratory droplets during extended periods of physical proximity, infected sores, body secretions, and common bedding or clothing [18]. In this light, healthcare professionals dealing with suspected or confirmed infected patients have higher chances of acquiring the infection since it can be transmitted through contaminated needle injury or skin contact with infected lesions or fomites and therefore require personal protective equipment (PPE), including gowns, gloves, face mask, face shield, and goggles.

Among the re-emergence of the monkeypox virus, data trends also show a probable sexual transmission hypothesized to be via direct skin-to-skin contact or through an exchange of genital secretions because of the presence of genital lesions in infected patients, since the majority of cases have been reported in individuals who identify as gay, bisexual, or, more precisely, MSM. Some patients may additionally have proctitis, in which case the clinical picture might get mistaken for a sexually transmitted infection (STI) especially if no preceding prodromal symptoms are present [19]. The recent monkeypox outbreak reports some cases with no symptoms other than genital lesions. Semen specimens collected from three males infected with monkeypox in Italy tested positive for the presence of the virus, strengthening the implication of sexual transmission of monkeypox in males [20,21].

An infected individual can transmit infection up to 21 days before the onset of any symptoms [12,19]. Misdiagnosis or delayed diagnosis results in increased dissemination of monkeypox infection as the infected individual is not correctly diagnosed and hence not isolated and sent home on antibiotics. Risk factors for the acquisition of monkeypox infection include age extremes such as children and elderly people, pregnancy, MSM sexual practices, immunocompromised states such as human immunodeficiency virus (HIV), and being a healthcare professional [12].

Although sufficient evidence exists in the literature that reports transmission of monkeypox infection from animals to humans and also among humans themselves, a unique case of human-to-animal transmission of the virus was reported in two men in Paris, who were inhabiting the same household under a polyandrous partnership, presented with anal ulceration and vesiculo-pustular body rash six days after having sex with other men; real-time PCR confirmed the diagnosis of monkeypox West African clade. Twelve days following their infectious manifestation, their domesticated dog, a male Italian and otherwise healthy greyhound, started to develop abdominal pustules with anal ulceration. A PCR test performed on the dog's sample revealed the monkeypox virus. The report described this case as the first ever known event of transmission of monkeypox infection from a human to an animal [22]. Additionally, pregnant ladies infected with monkeypox can communicate the infection to their fetus(es) through the placenta during pregnancy or via physical contact around birth, which can result in monkeypox infection in neonates [11,23]. 

A provisional diagnosis of monkeypox infection should be considered in patients with peri-genital rash, who have a recent travel history to affected areas, categorize as men who copulate with men, have a contact history with people having an identical rash, or those who are suspected or diagnosed cases of monkeypox infection [19]. However, it is still unknown if an asymptomatic infected person can pose a transmission risk to the general public [23]. It is hypothesized that immunity status and route of infection determine the level of disease severity. In the 2017 monkeypox epidemic in Nigeria, HIV-positive individuals exhibited greater morbidity with higher numbers of skin lesions, along with genital lesions than the HIV-negative population. In addition to this, invasive routes of infection that cause a breach of mucocutaneous membranes, such as an animal bite, are likely to cause disease with a shorter incubation period but with a more severe disease course (49.1% vs.16.7%) and higher chances of hospitalization (68.8% vs.10.3%) as compared to non-invasive modes of exposure, for instance, fomites [24-25].

Clinical features

Monkeypox manifests in a way similar to smallpox, but with a less severe course, starting with a prodromal phase, which includes fever, flu-like symptoms, headache, and myalgia with the addition of lymphadenopathy, which is absent in the latter [6,12,26]. A case series of 2022 monkeypox outbreaks from Portugal reported inguinal lymphadenopathy to be more common than cervical and axillary lymphadenopathy [27]. The mean latency period for symptoms to appear is stated to be 5-13 days or 5-21 days [12,19].

A maculopapular rash with centrifugal distribution usually follows 3-4 days after the prodrome. The rash may initially be peri-anal or peri-genital and then spread out to the trunk, extremities, and face. The rash appears on the face in 95% of the cases, on palms and soles in 75%, mucous membranes in 70%, genitalia in 30%, and cornea and conjunctiva in 20% of cases [11]. The infected lesions are well-circumscribed, with a central depression, embedded in deeper skin layers, and may be painful or itchy. They progress through various stages as macules (non-elevated discolored skin patch), papules (slightly raised skin lesions), vesicles (fluid-filled blisters), pustules (pus-filled blisters), and then eventually scabs or crusts that desiccate and later fall off. Other patients may get a rash first followed by flu-like symptoms or vice versa, or just the rash with no prodromal symptoms [19,28-29]. The infectious state starts with the symptomatic phase and ends after the desiccation of skin lesions and healing, which may take up to four weeks.

The current outbreak of monkeypox shows several atypical features as compared to the past, and the prodrome has milder symptoms, which may even go unnoticed in some cases until the appearance of rash later on; in MSM or people with peri-genital rash, even the skin lesions manifest a limited distribution [21,27,30-31]. Enanthem on oropharyngeal mucosa can impose pain and difficulty in oral intake. The disease severity and complications are directly proportional to the count of skin lesions [32].

HIV is common in the MSM population; therefore, HIV patients can also get infected with monkeypox regardless of their CD4 T lymphocyte count. In some cases, co-infection with syphilis also exists. Monkeypox in HIV patients usually starts with genital ulcers, and febrile prodrome is less common. Morbidity and mortality are higher in HIV patients with lower CD4 counts [33].

Complications include secondary bacterial infections that can cause bronchopneumonia, encephalitis, keratitis, diarrhea, and even sepsis [5] [11]. People unvaccinated against smallpox are also susceptible to superimposed bacterial infections of the skin [32]. 

Diagnosis

Differential diagnoses for monkeypox include chickenpox, measles, herpes, syphilis, scabies, and drug-induced allergy. Diagnosis is confirmed by polymerase chain reaction (PCR) [3,11]. Monkeypox testing services were initially available only at state health departments, but, eventually, testing was expanded to include commercial laboratory companies as well. Samples are collected from skin lesions and tested on PCR, and identification of monkeypox DNA confirms the diagnosis. CDC guidelines recommend the collection of multiple samples by vigorous swabbing or brushing of the lesion. Two aseptic swabs made of polyester or Dacron are used for sample collection; next, the applicator of each swab is broken and placed into a capped tube with an O-ring, or they are placed in two separate sterile containers, and no media should be added to these. In case of a positive confirmatory diagnosis, all contacts within the symptomatic period should be tracked and observed for any signs and symptoms for at least 21 days from the last exposure to the infected individual [5,19].

Scabs, swabs, and aspirated fluids from lesions are preferred over blood samples for PCR testing due to shortened lifespan of the virus in the blood. These samples require room temperature, but no media for transportation, except for tissue biopsies, which require transportation in a frozen state on dry ice. Histopathology findings show ballooning and multinucleation of keratinocytes with full-thickness epidermal necrosis, nuclear enlargement with an eosinophilic center and basophilic halo, and orthopoxvirus-specific eosinophilic cytoplasmic inclusions, called "Guarnieri bodies" or "B-type inclusions" [34-35]. Immunohistochemistry can also identify antigens on biopsies, while the modality of PCR can even differentiate between the two monkeypox clades. Serology has a limited diagnostic utility, as it cannot differentiate between different orthopoxviruses due to cross-immunological reactivity, but antibody titers can be measured to check vaccine responses. In addition, these contacts cannot donate blood, organ, or bone marrow within this 21-day time window [12].

One would typically expect a rising pattern in the total leucocyte count (TLC) in the case of bacterial sepsis. However, a decline in the differential count of neutrophils or neutropenia can occur in fulminant monkeypox infections with bacterial sepsis and is related to higher morbidity and mortality [36]. Therefore, it is important to rule out any coexisting bacterial sepsis and treat it accordingly.

Key definitions

The following provisional definitions have been proposed by the European Centre for Disease Prevention and Control (ECDC) and CDC (please see Table 2) [12,37].

Patients who fit the definition of a probable or suspected case should undergo a monkeypox-specific PCR or an orthopox-specific PCR, followed by confirmation by nucleotide sequencing. If these tests come out to be negative, these patients should be excluded [12].

**Table 2:** Key definitions of monkeypox cases by the European Centre for Disease Prevention and Control (ECDC) and Centers for Disease Control and Prevention (CDC) This table quotes the exact case definitions from the websites of ECDC and CDC and does not require copyright permission as per the websites [12,37].

	Suspected Case/Probable Case	Confirmed Case
European Centre for Disease Prevention and Control (ECDC)	“A probable or suspected case of monkeypox has an unexplained generalized or centrifugally spreading localized maculopapular or vesiculopustular rash with central depression or scabbing, lymphadenopathy and one or more other monkeypox symptoms (including but not limited to fever usually >38.5°C, headache, back ache, fatigue, lymphadenopathy) or an unexplained rash on any body part in addition to one or more monkeypox symptoms with an onset from or after 01 March 2022 and one of the following: an orthopox specific positive PCR without further testing for nucleotide sequencing, electron microscopy, serology etc, an exposure to suspected or confirmed monkeypox case(s) within 21 days prior to the onset of symptoms, a travel history to monkeypox endemic countries within 21 days prior to the onset of symptoms, a history of multiple or anonymous sexual partners within 21 days prior to the onset of symptoms, irrespective of the sexual orientation, men who have sex with men (MSM)” [12].	“Confirmed by laboratory tests, that is, either a monkeypox specific positive PCR or an orthopox virus specific positive PCR which further detects monkeypox virus on nucleotide sequence determination” [12].
Centers for Disease Control and Prevention (CDC)	Suspected Case: “New characteristic rash OR meets one of the epidemiologic criteria and has a high clinical suspicion for monkeypox.” Probable Case: "No suspicion of other recent Orthopoxvirus exposure (e.g., Vaccinia virus in ACAM2000 vaccination) AND demonstration of the presence of Orthopoxvirus DNA by polymerase chain reaction of a clinical specimen OR Orthopoxvirus using immunohistochemical or electron microscopy testing methods OR Demonstration of detectable levels of anti-orthopoxvirus IgM antibody during the period of 4 to 56 days after rash onset” [37].	“Demonstration of the presence of monkeypox virus DNA by polymerase chain reaction testing or Next-Generation sequencing of a clinical specimen OR isolation of monkeypox virus in culture from a clinical specimen” [37].

Molecular pathogenesis

Monkeypox is oblong or brick-shaped, enveloped DNA virus, which is slightly pleomorphic, with a biconcave-shaped nucleic acid core and two lateral bodies, and an overall size of 200-400 nm. Its genome, despite being a double-stranded DNA, measures 197 kilo-base-pair linearly, with around 200 genes; the viral life cycle occurs in the cytoplasm of host cells [38-39]. Like all orthopoxviruses, its genome comprises two terminal ends, called telomeres. These contain identical but oppositely oriented adjacent repetitive nucleotide sequences, called inverted terminal repeats (ITRs); they make up about 3% of the monkeypox viral genome and are responsible for genetic variation and mutations [40]. The monkeypox genome encodes several essential viral proteins that are categorized into three groups: i) viral proteins that assist the virus to invade host cells by attaching to their glycoprotein receptors and entering via macropinocytosis; ii) viral proteins that liberate intracellularly proliferated viral replicas into the extracellular environment; and iii) proteins that serve as immune modulators to host cell defenses [41].

Poxviruses, including monkeypox, are proportionately larger than other viruses. Hence, they cannot breach mechanical barriers in the target cell by passage through host cell gap junctions The relatively large size of monkeypox alerts the host immune system easily and early on; hence, the monkeypox virus needs an extensive strategy to invade and survive within the host cells. In order to evade the host's immune system, the virulence genes in the monkeypox virus encode proteins that modulate host immune responses to facilitate viral survival inside the host. These immunomodulating proteins are further divided into three categories: virotransducer proteins, virostealth proteins, and viromimic proteins. The former two work intracellularly, while the latter works extracellularly. The virotransducer proteins interfere with the host's immune responses to monkeypox infection by inhibiting innate antiviral signaling pathways that involve oxidative burst and apoptosis [42-43]. The virostealth proteins impede viral detection by the infected host’s immune system through inhibition of its antigen recognition receptors and cells, such as the major histocompatibility complex class 1 (MHC 1) and CD4+ [44]. This reduces cell-mediated immunity and impairs the cytotoxic T-cell-mediated annihilation of the virus-infected cells. The viromimic proteins are of two types, viro-receptors and virokines. The viro-receptors are encoded by the viral genome, express as glycoprotein receptors on the host cells, and attach host cytokines and chemokines to themselves, thus preventing their bonding to the original target receptors and subsequent functioning. Meanwhile, the virokines imitate reservoir’s cytokines and chemokines, dysregulating their physiological operations. All these immunomodulatory factors in monkeypox work synergistically to elude host immune responses and facilitate viral replication [41,45-46].

Following its entry into the reservoir cell by macropinocytosis and fusion, the virus starts to uncoat and release viral genome in the host cell’s cytoplasm where this viral DNA replicates to generate more viral entities, along with viral genomic transcription and translation to produce viral proteins, which modulate the host immune system [47]. From the entrance site, the infection then extends to regional lymph nodes. Next, it reaches the bloodstream through lymphatic drainage, and primary viremia occurs, which coincides with the incubation period, conventionally ranging from 7 to 17 days. Primary viremia results in dissemination to other organs by circulation; this is called secondary viremia, which coincides with the prodromal period and usually lasts 1-4 days. The secondary viremia coincides with the prodromal phase, which lasts 1-4 days, followed by skin rash and other symptoms typical of the monkeypox infection [48-49]. The virus invades cutaneous blood vessels preceding the development of skin rash. The mechanism through which the virus ascends to the superficial avascular planes of the skin is not exactly known, but it is hypothesized that dermal macrophages, such as Langerhans cells, are involved in it, as this is the known mechanism for vaccinia virus infection. This explanation sounds convincing because monkeypox-infected skin pustules show an influx of resident antigen-presenting cells and CD3+ T cells. Eventually, enanthem erupts on mucous membranes including the oropharynx as well, which then transform into ulcers and shed virus in the saliva [38,50].

Genomic analysis of the two monkeypox clades, the Central African clade (alternatively called Congo Basin clade) and the West African clade, reveal genes that determine virulence in the respective clades. Open reading frames of the West African clade contain deletions and fragmentations, which result in a relative decrement in its pathogenicity as opposed to the other clade [51-52]. A study done in Wisconsin, USA, during the 2003 monkeypox outbreak by Hammarlund et al. revealed that antiviral cytotoxic and helper T-cells could perceive monocytes infected with Vaccinia virus and, in response, produced inflammatory cytokines, such as interferon-gamma (IFN-γ) and tumor necrosis factor-alpha (TNFα), but could not generate the same immune response to monkeypox-infected monocytes. The inhibition of cytotoxic T-cell response against monkeypox-infected monocytes was found to be mediated by the impaired receptor-mediated T-cell activation; unlike the other Orthopox virus, cowpox, no interference in the MHC expression was observed. The study inferred that the monkeypox virus produces immunomodulating proteins, which suppress immune responses by the host’s T-cells [53]. A gene that encodes a protein called complement control protein (CCP) is unique to the Central African clade and deficient in the West African clade due to deletions in open reading frames. This protein impairs both classical and alternative pathways of the complement system and is reportedly one of the multiple immunomodulating factors that are responsible for higher virulence in the Central African clade [54-55]. 

Kindrachuk et al. proposed that the Congo Basin clade modulates host cell responses differently than the West African clade, since the former manifests increased disease severity with a higher case fatality rate. Their experiment demonstrated that the Central African clade downregulates apoptosis in infected cells. Moreover, different patterns of phosphorylation were observed in the two clades, which were proven as potential targets for pharmacological intervention, including excessive Akt S473 phosphorylation and deficient p53-Ser15 phosphorylation. The impedance of Akt S473 phosphorylation by pharmacologic intervention led to a 261-fold decline in the Central African clade yield; however, the West African monkeypox clade remained unaffected [56].

Genetic analysis of the Central African strain (ZAI-96) and three West African strains (SL-V70, COP-58, and WRAIR-61) disclosed a difference of 0.55-0.56% in the nucleotides between the Central and West African strains, with 173 and 171 unique functional genes, respectively [57]. 

Novel mutations

Monkeypox is a DNA virus, so it does not exhibit multiple novel mutations, such as RNA viruses, for instance, HIV or SARS-CoV-2 [58]. Nevertheless, monkeypox viral strains, procured during the 2022 outbreak, are reported to have undergone multiple, about 40, mutations so far. In typical evolutionary timelines, a microbe would be expected to undergo these many mutations in about 50 years. Rampant transmission among humans has been hypothesized as a reason for monkeypox mutations. In evolution, pathogens usually undergo such mutations to adapt better against emerging sustainability threats, but, sometimes, harmless mutations occur as well. The exact mechanism behind the development of such mutations is unknown, but the literature suggests that certain enzymes in the host's immune system induce mutations in them if they encounter viruses [59].

A systemic analysis by Wang et al. of the ongoing, widespread monkeypox epidemic in historically non-endemic countries for this infection revealed that 2022 strains phylogenetically belong to the same lineage as 2018 strains. However, the strains from 2022 contain 46 new consensus mutations, inclusive of 24 nonsynonymous mutations. If enough of such mutations are triggered in this virus, this can get more detrimental, since nonsynonymous mutations change protein sequences and favor evolutionary progression via natural selection. A total of 187 proteins were found to be encoded by the mutations, of which 10 proteins were more susceptible to mutations. The exact effect of these mutations on viral functionality is still unknown [60]. Based on the literature, the first-ever case of transmission of monkeypox among humans was known in 2018. Since then, the monkeypox virus has exhibited a mutation rate that is tenfold its standard mutation rate. Currently, sufficient knowledge does not exist about the consequences of these mutations, but the rate is alarming to scientists and warrants more research into the area [61].

Prevention

Variola, cowpox, vaccinia, and monkeypox virus all belong to the Orthopox genus. Immunological cross-reactivity and cross-protection exist among the Orthopox species, and hence infection with any one of these species provides some extent of protection against the others [62]. It has been found that vaccination against smallpox offers immunological protection against all orthopoxviruses, including monkeypox. The termination of smallpox vaccination due to the global elimination of smallpox after 1980 is hypothesized to instigate rampant re-eruption of the monkeypox infection with a twentyfold increase in the incidence reported in 2010 than that in the 1980s [63].

A live attenuated vaccine of Vaccinia virus with genetic modification, Ankara-Bavarian Nordic (MVA-BN strain), has been developed under brand names "JYNNEOS" and "ACAM2000®" in the USA, "IMVANEX" in Europe, and IMVAMUNE in Canada against smallpox and monkeypox [64]. The Food and Drug Administration (FDA) approved JYNNEOS in 2019 for prophylaxis of smallpox and monkeypox in high-risk groups, aged 18 years and above [65]. The recommended dosing for JYNNEOS is two subcutaneous doses of 0.5 ml, with an interval of four weeks for the first smallpox vaccination. Individuals previously vaccinated receive only one dose [66]. ACAM2000® is administered intradermally as a single dose through multiple punctures [67]. Historical data show Vaccinia virus vaccination to be 85% effective against monkeypox [68]. Vaccinated individuals have significantly lower chances of being infected, and, even if they do get infected, the disease morbidity is markedly reduced. 

**Figure 2:**
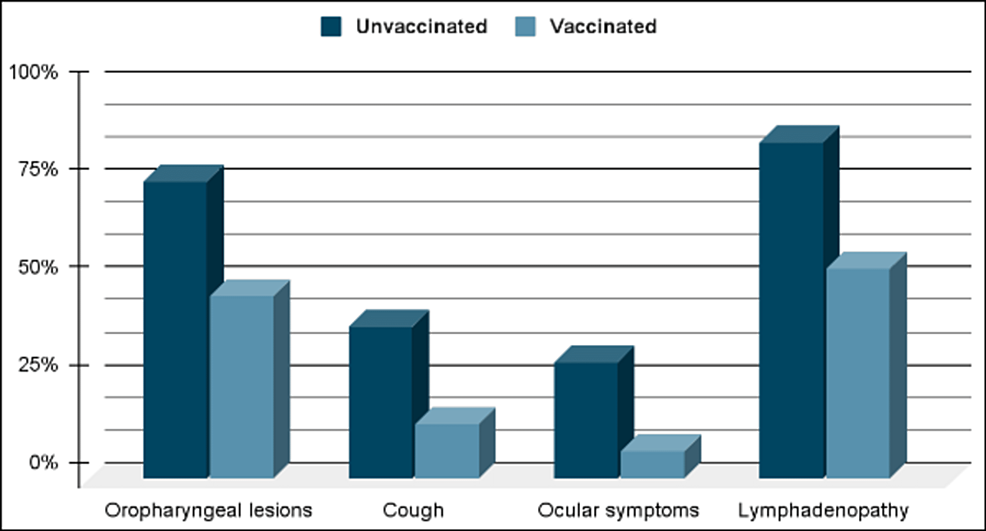
Frequency of monkeypox symptoms in smallpox vaccinated vs. unvaccinated groups The figure is a graphical representation, created by authors and based on data available in the literature [24].

The percentage of various symptoms in smallpox-vaccinated versus unvaccinated groups is depicted in Figure 2 [24].

Smallpox vaccination can be given prophylactically to prevent monkeypox infection before or after a suspected or confirmed viral exposure. Pre-exposure prophylaxis (Pre-EP) is indicated only in high-risk groups, such as immunocompromised persons or medical personnel who are frequently exposed to orthopoxviruses; it is indicated when prolonged high exposure contact has taken place. Exposures that warrant a Pre-EP include unprotected direct mucocutaneous exposure to infected patient’s skin, body fluids, contaminated fomites, or standing with no personal protective equipment within 6 feet radius of an infected patient during any procedure that may produce aerosol from patient’s secretions, body fluids, and dry exudates or presence within 6 feet of an unmasked patient for at least three hours [64]. The CDC recommends post-exposure prophylaxis (Post-EP) to be given within four days of exposure. A Post-EP given between four and 14 days after exposure reduces the disease severity but does not protect from the infection incidence in the first place [69]. 

Sexual transmission has been implicated in the Vaccinia virus following its administration as the smallpox vaccine and multiple cases have been reported so far. In one such case, a military man who was recently vaccinated with the smallpox vaccine removed his bandages covering the vaccination site and had unprotected sexual intercourse following digital vaginal contact. His female partner had atopic dermatitis and developed a vulvar lesion four days after this event, which tested positive for the Vaccinia virus on PCR assay [70]. In another case, a painful perianal rash and an upper lip lesion were reported in a man who had unprotected sexual intercourse with another man who had been recently vaccinated with smallpox and had his vaccination site uncovered. This second contact, while experiencing a perianal rash, had sexual contact with a third man who developed papular lesions on his penis, along with prodromal symptoms two days following this sexual contact. In both patients, testing of the lesions confirmed the presence of non-variola Orthopoxvirus [71].

No doubt that there is sufficient literary evidence for the utility of the smallpox vaccine for Pre-EP and Post-EP, but care must be taken for not leaving the vaccination site uncovered and covering it with a bandage until the scab that forms after vaccination falls off on its own, which may take two to three weeks due to risk of viral shedding from the vaccine site and possible transmission to immunocompromised or atopic individuals. Other precautions for this include changing the bandage every one to two days, proper disposal of the removed bandages, and proper hand sanitization while handling the wound and bandages [72]. Another study demonstrated the role of topical application of povidone-iodine ointment in reducing the risk of contact spread from the vaccine site, from seven days post-vaccine administration to complete skin healing [73].

Treatment

Monkeypox infection conventionally exhibits mild clinical manifestations, and the majority of patients can recuperate without any treatment; however, prompt diagnosis and treatment are still crucial as it is a contagious infection with extensive outbreaks in many countries and can otherwise turn into a pandemic. Testing suspected individuals, isolation and treatment of confirmed cases, contact tracing, use of PPE in healthcare workers, and reporting new cases to local public health authorities can limit infection and transmission rates. Morbidity is higher in elderly and immunocompromised groups, the current global CFR as of July 19, 2023, is about 0.172% (please see Table 1 ) [14]. Prognosis is good in immunocompetent groups, especially with antivirals and supportive treatment [12,14].

As per the CDC guidelines "Medical Countermeasures Available for the Treatment of Mpox," no specific treatment is currently approved for monkeypox infection; however, antivirals such as tecovirimat, which should be the first line of treatment, followed by brincidofovir, cidofovir, and vaccinia immune globulin (VIG), can be beneficial. Treatment options should only be considered for high-risk groups; these include immunocompromised or people having comorbidities, elderly, pregnant or breastfeeding individuals, children younger than eight years of age, people having skin conditions that cause a breach in mucocutaneous barriers, and patients experiencing severe symptoms for monkeypox infection that may or may not require hospitalization. Treatment options for monkeypox are an area that still requires extensive research as the utility of certain antiviral medications in treating monkeypox infection is still controversial and debatable. The evidence available in the literature is preclinical with an insignificant amount of data available on human disease models [74]. Literature proves the effectiveness of smallpox vaccination for Pre-EP and Post-EP; antiviral drugs (tecovirimat and brincidofovir) and VIG have shown promising results in active infections. Tecovirimat and brincidofovir are licensed by the United States FDA for the treatment of smallpox. Meanwhile, cidofovir is FDA-approved for the treatment of CMV retinitis in patients with AIDS. These antivirals are now being explored as possible treatment options for monkeypox infection [50,61].

Tecovirimat, a 4-trifluoromethyl phenol derivative antiviral (also known as ST-246 or TPOXX®®), has now been approved by the European Medicines Agency (EMA) for the treatment of monkeypox. Although a variety of animal species models have demonstrated the therapeutic effectiveness of Tecovirimat against all Orthopox virus infections, no significant data are available in the literature to establish its efficacy in humans; however, a clinical trial called STOMP is currently under progress to assess it [74-75]. By targeting the Orthopox virus F13L gene that encodes VP37 membrane protein, the drug prevents enveloping of intracellular mature viral particles that would otherwise disseminate outside the infected cell into the host’s body [76-77]. Tecovirimat shows an established safety profile in humans at an oral dosage of 600 mg twice daily, and headache and nausea are the two most commonly reported adverse effects [75]. Different NHP models from multiple studies show that tecovirimat reduces morbidity and mortality in monkeypox infection, and its maximum effect is seen when administered within five days of the infection. In real scenarios, the symptoms appear much later following the onset of infection, and this suggests a decrease in the effectiveness of tecovirimat in treating symptomatically active monkeypox infection than when given as Post-EP. Smallpox vaccine alone was not found to be much effective for Post-EP, as it only reduces the disease severity but does not prevent it, but its combination with oral tecovirimat or oral administration of tecovirimat alone was found to be 100% protective against the infection [78-81]. Apart from NHP models, certain studies provide evidence of the effectiveness of tecovirimat in human models as well. A case series of monkeypox-infected humans from Massachusetts, USA, reported markedly reduced disease severity and resolution of symptoms around day five, nine, or 14 when they were administered tecovirimat 600 mg orally, every 12 hours for two weeks during the active symptomatic phase of the infection [82]. 

Tecovirimat can be administered either orally or intravenously (IV). Fecal elimination is the major clearance route, and no fetal risks have been reported with gestational usage in animal models. Some major adverse reactions reportedly include headache, gastrointestinal upset, xerostomia, and hypersensitivity reactions. The recommended dosage regimen for monkeypox treatment is 14 days in animal models and 21 days in safety data, and further clinical trials are still ongoing to explore 28-day regimens. See Table 3 for dosage recommendations [83].

**Table 3:** Weight-adjusted daily dosage for oral and intravenous (IV) tecovirimat The table is the authors' own creation, based on the data available in the literature [83].

	Body weight	Strength	Dosing
Oral Tecovirimat	13-24 kg	200 mg	12 hourly
Oral Tecovirimat	25-39 kg	400 mg	12 hourly
Oral Tecovirimat	40-119 kg	600 mg	12 hourly
Oral Tecovirimat	≥ 120 kg	600 mg	8 hourly
Intravenous (IV) Tecovirimat	3-34 kg	6 mg/kg, over hours	12 hourly
Intravenous (IV) Tecovirimat	35-119 kg	200 mg, over 6 hours	12 hourly
Intravenous (IV) Tecovirimat	≥ 120 kg	300 mg, over 6 hours	12 hourly

An acyclic nucleoside phosphate, cidofovir, and its lipid-conjugated prodrug, brincidofovir (also known as CMX001), are antivirals that block viral DNA synthesis of orthopoxviruses, including monkeypox virus, by inhibiting the viral DNA polymerase. Cidofovir is a monophosphate nucleotide and needs to be phosphorylated by intracellular kinases to get activated. In contrast, brincidofovir is cleaved following its intracellular uptake to release the phosphorylated cidofovir molecule already present in the drug. This explains brincidofovir’s higher cellular toxicity and stronger antiviral activity, greater selective index, and 25-fold efficacy than cidofovir against monkeypox, vaccinia, variola, and cowpox viruses [76]. Brincidofovir has exhibited antiviral activity against monkeypox in animal models [84-85].

Brincidofovir is available in oral formulation only; 51% of the drug undergoes urinary clearance, while 50% of it is excreted in feces. The risk of teratogenicity has been reported in animal models, and partners should use contraception during and after the treatment till at least four months after the last dose. Nausea, vomiting, diarrhea, abdominal pain, and elevation of liver enzymes are the major known adverse effects. Brincidofovir is recommended to be taken in two doses, one week apart (days one and eight), with the following weight-adjusted dosage: “<10 kg: 6 mg/kg (suspension); 10 kg to <48 kg: 4 mg/kg (suspension); and 48 kg and above: 200 mg (20 mL or one tablet)” [83].

Cidofovir is available in IV formulation only. It is known to be teratogenic, hence is not recommended in pregnant patients. Up to 75-80% of the drug undergoes renal clearance. Adverse effects include neutropenia, decreased ocular pressure, and nephrotoxicity. Limited data are available on its dosage regimen, but the available literature recommends a single dose of 5 mg/kg. About 2 gm probenecid is given three hours prior to cidofovir to prevent potential nephrotoxicity, followed by 1 L 0.9% NS prior to administration of 5 mg/kg cidofovir, diluted in 100 ml NS, and infused IV over 1 hour. If a higher volume can be tolerated, an additional 1 L NS can be given over one to three hours, starting with cidofovir infusion. Finally, 1 g probenecid should be repeated two and eight hours post-cidofovir infusion. The goal is to reduce the nephrotoxic potential of cidofovir by the administration of probenecid to reduce the tubular secretion of the antiviral along with good IV hydration to reduce the renal contact time [83,86].

Among the aforementioned three antiviral drugs, tecovirimat is considered the first-line and the best treatment option for monkeypox infection because of an established safety profile in humans, great antiviral response in both prophylactic and therapeutic terms, broad antiviral spectrum, and a more tolerable adverse effects profile [78,87]. VIG can treat complications of vaccinia/smallpox vaccination, such as eczema vaccinium, severe generalized vaccinia, etc., when given intravenously. However, it is neither approved by CDC or EMA. No significant literary evidence exists to demonstrate the efficacy of VIG in monkeypox infection. However, a healthcare provider may decide to administer it to severely immunocompromised individuals with impaired T cell responses, conditions which contraindicate the usage of smallpox vaccination for Post-EP to monkeypox [74].

Limitations

We have quoted the latest global statistics available for the number of cases and deaths; however, they are being updated every two weeks on the CDC website, and this can be a limitation to the current statistics mentioned in our review article.

## Conclusions

Monkeypox has now become a globally emerging public health challenge, with low statistics in historically endemic areas and rampant growth throughout non-endemic areas. Global cessation of the smallpox vaccine has been hypothesized to be the cause of recurrent monkeypox outbreaks across various parts of the globe, from time to time. Previous outbreaks outside the endemic areas were usually limited to single countries, but the ongoing 2022 outbreak has hit multiple countries in the Western Hemisphere simultaneously. Many atypical features have been observed in this outbreak, including sexual transmission among men who copulate with men, along with genital rash. Two clades with several mutated strains have been identified so far. The current outbreak is of the West African clade, which is less virulent than the Central African clade. The infection is usually self-limiting, with low death tolls; the current global CFR is about 0.712%. 

Like any other viral illness, the treatment is mainly symptomatic; however, severely immunocompromised groups or those developing severe complications can be administered antiviral drugs such as tecovirimat, brincidofovir, and cidofovir. The FDA and CDC have recommended the smallpox vaccine for Pre-EP and Post-EP of monkeypox as it significantly reduces morbidity and mortality rates and is now commercially available in the USA, Europe, and Canada. These treatment options have shown promising results, but further research is still needed in this area to establish solid treatment guidelines in order to abolish the current monkeypox outbreak.
